# Supplementary material for: Predicting CTCF-mediated chromatin interactions by integrating genomic and epigenomic features
Source: Nat Commun. 2018 Oct 11;9:4221. doi: 10.1038/s41467-018-06664-6 (PMC6181989; doi:10.1038/s41467-018-06664-6)
Supplement: Supplementary file 1 — Supplementary Information [file 41467_2018_6664_MOESM1_ESM.pdf]

Predicting CTCF-mediated chromatin interactions by integrating genomic and epigenomic features

Supplementary Information

Kai et al.

Supplementary Figure 1

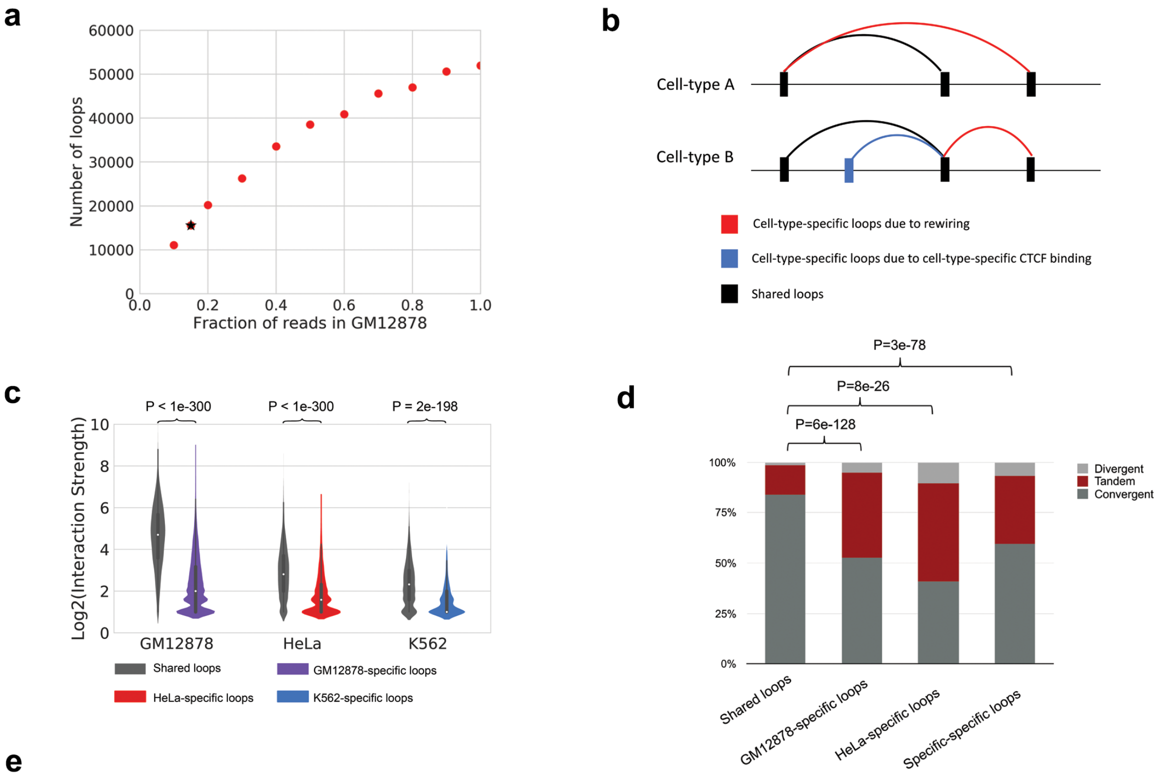

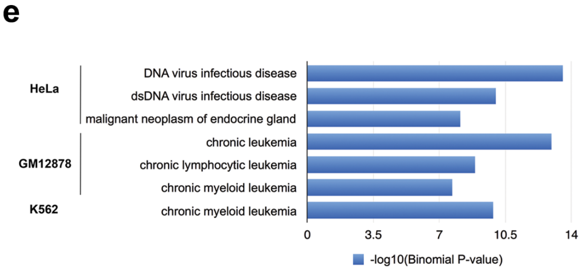

g

Comparison	Type	Associated with cell-type-specific loops	Not associated with cell-type specific loops	Chi-square	P-value
HeLa-K562	DEGs	1999	1230	95.0	1.9e-22
	Non-DEG	1609	1620		
GM12878-HeLa	DEGs	3651	645	81.4	1.8e-19
	Non-DEG	3323	973		
GM12878-K562	DEGs	3128	593	63.1	2.0e-15
	Non-DEG	2855	866		

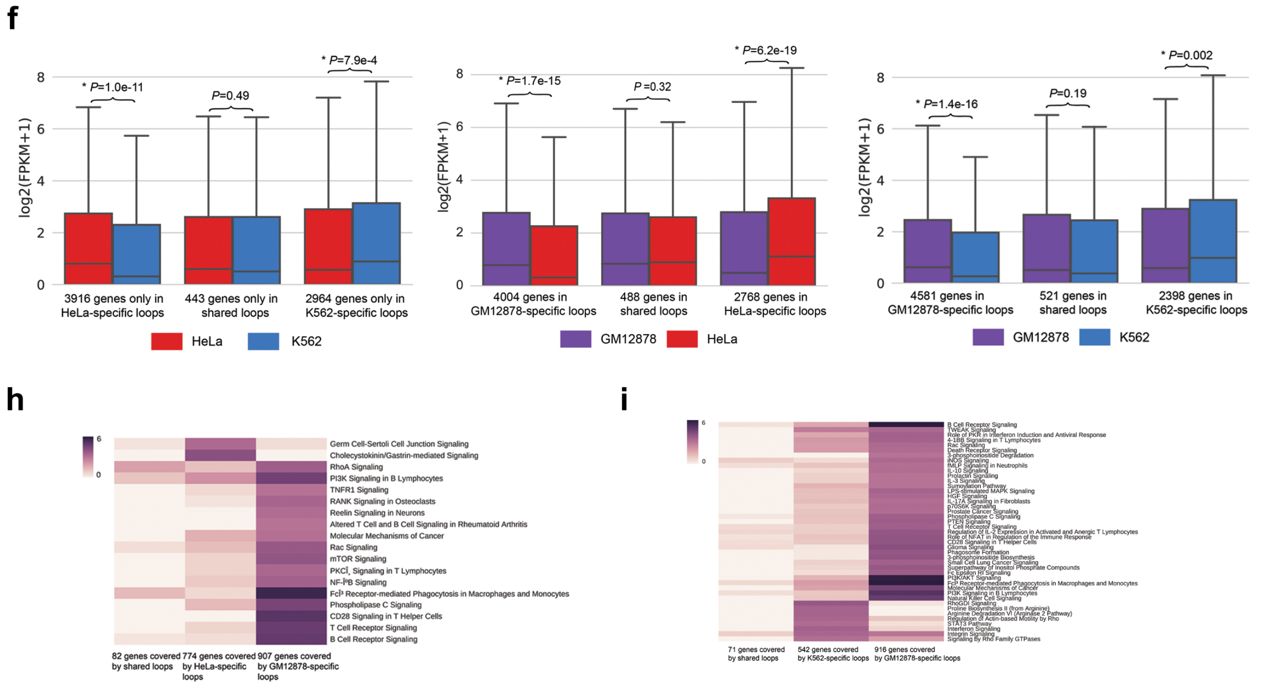

Supplementary Figure 1. CTCF-mediated loops exhibit cell-type-specificity.

(**a**) Down-sampling analysis of the GM12878 ChIA-PET data. The star denotes the point for 15% down-sampling.

(**b**) Illustration of two types of cell-type-specific loops.

(**c**) Violin plots show that shared CTCF-mediated loops are stronger than cell-type-specific loops. Interaction strength is defined as the number of Paired-End Tags (PETs) connecting the anchors. Shown at the center of the violin plot are miniature box plots, with centers representing the median value and boxes representing the first and third quartiles. *P*-values were calculated using Mann Whitney U test.

(**d**) Stacked bar plot comparing the pattern of motif orientation between cell-type-specific and shared loops. The *P*-values were calculated using Chi-square test.

(**e**) Disease Ontology analysis of Super-Enhancers (SEs) using GREAT reveals the disease origin of the three cell-types.

(**f**) Boxplots showing expression levels of genes associated with cell-type-specific and shared loops in pair-wise comparison. A gene is associated with a loop if its promoter region (TSS +/- 2kb) is inside the loop. *P*-values were calculated using Mann-Whitney U test.

(**g**) Contingency table for the number of loops associated with Differentially Expressed Genes (DEGs) and Non-DEGs among the three cell lines. Pair-wise comparison is shown.

(**h-i**) Canonical pathway enrichment analysis of DEGs associated with cell-type-specific and shared loops in (**h**) HeLa-GM12878 and (**i**) K562-GM12878 comparison. Color represents the -log10 (*P*-value).

Supplementary Figure 2

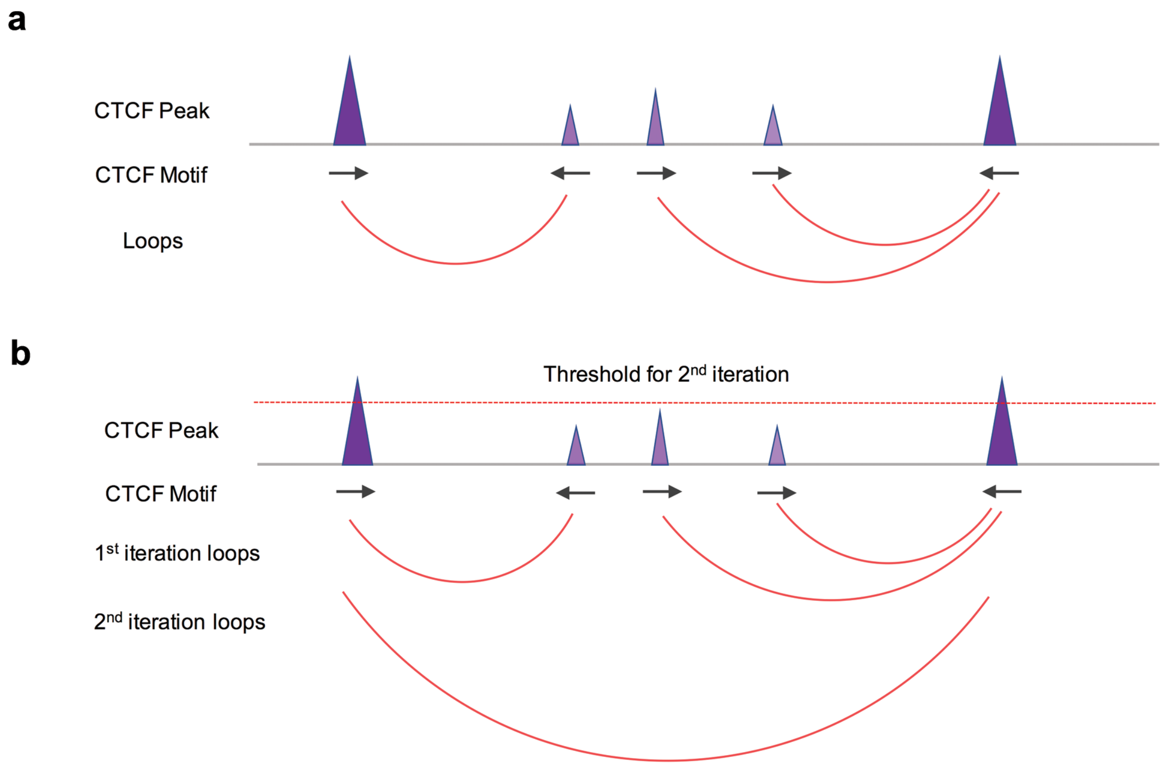

Supplementary Figure 2. Illustration of the naïve and Oti method.

(**a**) Illustration of the naïve method. This method pairs a CTCF-bound motif that resides on the forward strand to the nearest downstream CTCF-bound motif that resides on the reverse strand.

(**b**) Illustration of the Oti method. This method constructs loops in iterations by increasing the threshold of CTCF binding intensity. In each iteration, CTCF-bound motifs whose binding intensity are above the threshold were chosen, and naïve method was applied to construct loops. The loops constructed in different iterations are pooled together for the eventual result.

Supplementary Figure 3

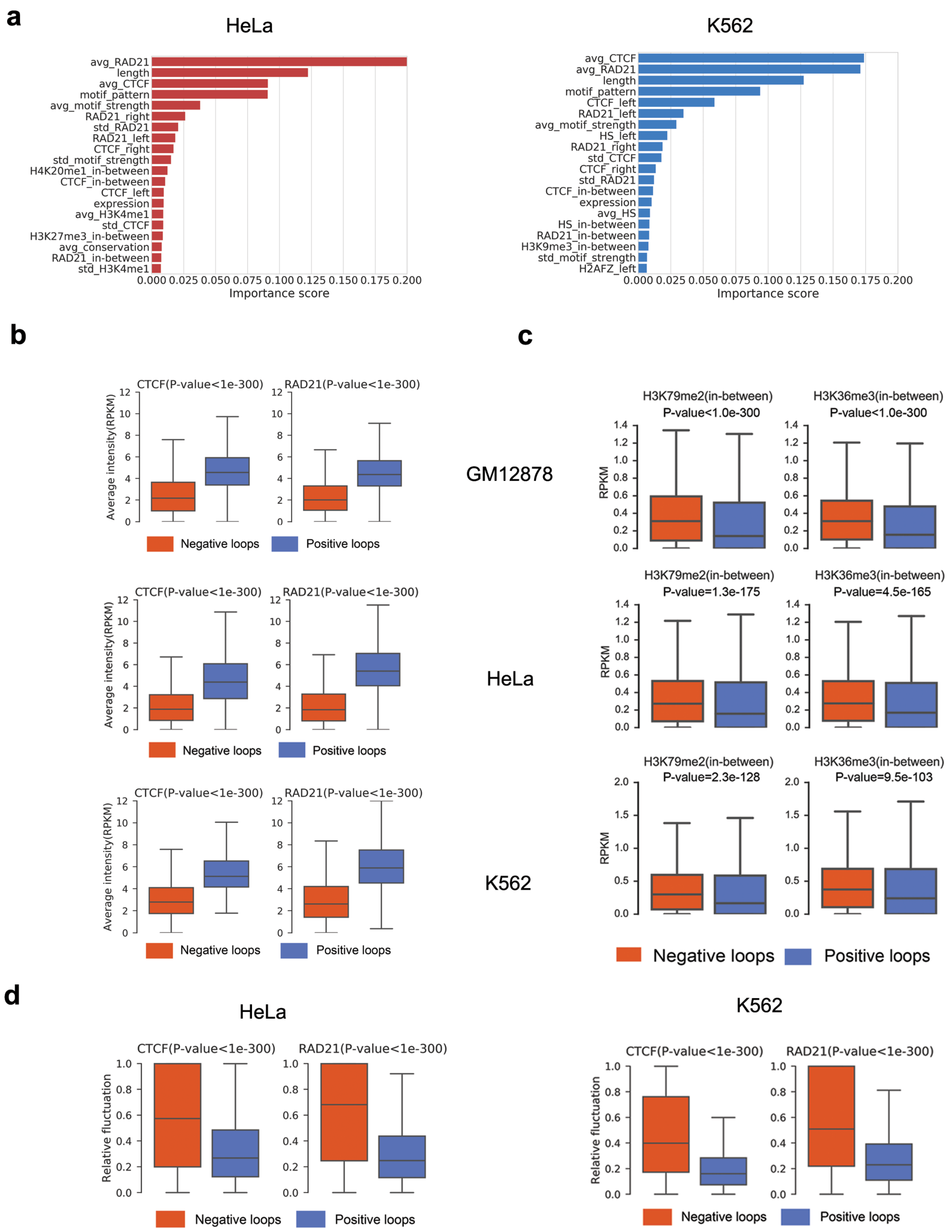

e

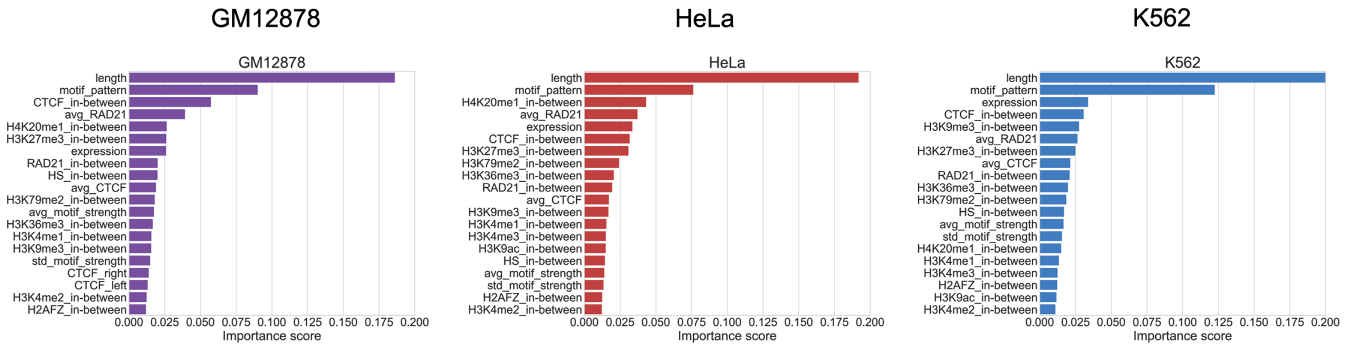
GM12878
HeLa
K562
GM12878
length
motif_pattern
CTCF_in-between
avg_RAD21
H4K20me1_in-between
H3K27me3_in-between
expression
RAD21_in-between
HS_in-between
avg_CTCF
H3K79me2_in-between
avg_motif_strength
H3K36me3_in-between
H3K4me1_in-between
H3K9me3_in-between
std_motif_strength
CTCF_right
CTCF_left
H3K4me2_in-between
H2AFZ_in-between
0.000 0.025 0.050 0.075 0.100 0.125 0.150 0.175 0.200
Importance score
HeLa
length
motif_pattern
H4K20me1_in-between
avg_RAD21
expression
CTCF_in-between
H3K27me3_in-between
H3K79me2_in-between
H3K36me3_in-between
RAD21_in-between
avg_CTCF
H3K9me3_in-between
H3K4me1_in-between
H3K4me3_in-between
H3K9ac_in-between
HS_in-between
avg_motif_strength
std_motif_strength
H2AFZ_in-between
H3K4me2_in-between
0.000 0.025 0.050 0.075 0.100 0.125 0.150 0.175 0.200
Importance score
K562
length
motif_pattern
expression
CTCF_in-between
H3K9me3_in-between
avg_RAD21
H3K27me3_in-between
avg_CTCF
RAD21_in-between
H3K36me3_in-between
H3K79me2_in-between
HS_in-between
avg_motif_strength
std_motif_strength
H4K20me1_in-between
H3K4me1_in-between
H3K4me3_in-between
H2AFZ_in-between
H3K9ac_in-between
H3K4me2_in-between
0.000 0.025 0.050 0.075 0.100 0.125 0.150 0.175 0.200
Importance score

f

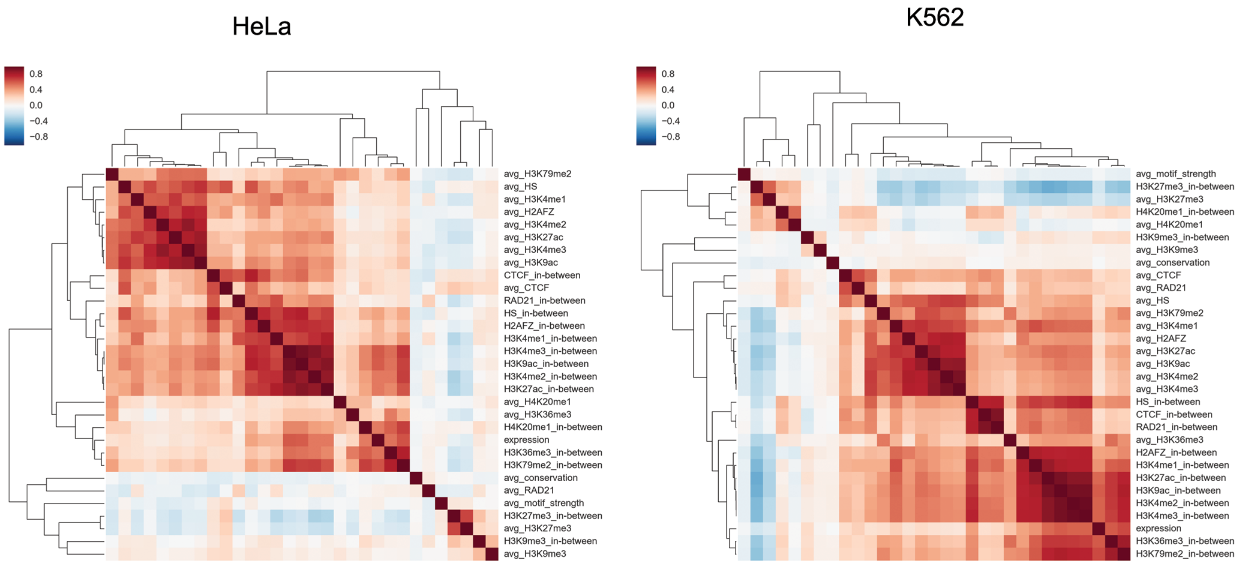
HeLa
K562
0.8
0.4
0.0
−0.4
−0.8
avg_H3K79me2
avg_HS
avg_H3K4me1
avg_H2AFZ
avg_H3K4me2
avg_H3K27ac
avg_H3K4me3
avg_H3K9ac
CTCF_in-between
avg_CTCF
RAD21_in-between
HS_in-between
H2AFZ_in-between
H3K4me1_in-between
H3K4me3_in-between
H3K9ac_in-between
H3K4me2_in-between
H3K27ac_in-between
avg_H4K20me1
avg_H3K36me3
H4K20me1_in-between
expression
H3K36me3_in-between
H3K79me2_in-between
avg_conservation
avg_RAD21
avg_motif_strength
H3K27me3_in-between
avg_H3K27me3
H3K9me3_in-between
avg_H3K9me3
0.8
0.4
0.0
−0.4
−0.8
avg_motif_strength
H3K27me3_in-between
avg_H3K27me3
H4K20me1_in-between
avg_H4K20me1
H3K9me3_in-between
avg_H3K9me3
avg_conservation
avg_CTCF
avg_RAD21
avg_HS
avg_H3K79me2
avg_H3K4me1
avg_H2AFZ
avg_H3K27ac
avg_H3K9ac
avg_H3K4me2
avg_H3K4me3
HS_in-between
CTCF_in-between
RAD21_in-between
avg_H3K36me3
H2AFZ_in-between
H3K4me1_in-between
H3K27ac_in-between
H3K9ac_in-between
H3K4me2_in-between
H3K4me3_in-between
expression
H3K36me3_in-between
H3K79me2_in-between

g

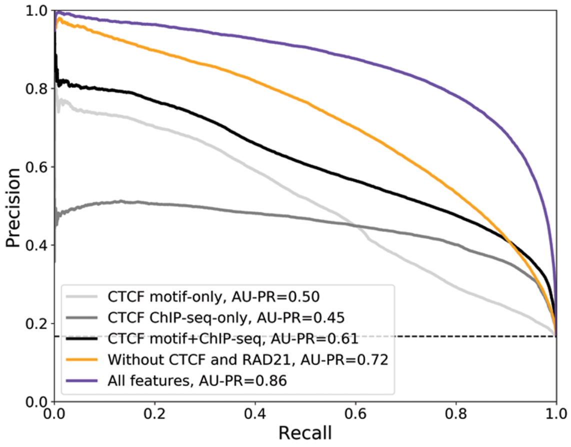
Precision
Recall
0.0
0.2
0.4
0.6
0.8
1.0
CTCF motif-only, AU-PR=0.50
CTCF ChIP-seq-only, AU-PR=0.45
CTCF motif+ChIP-seq, AU-PR=0.61
Without CTCF and RAD21, AU-PR=0.72
All features, AU-PR=0.86

Supplementary Figure 3. Results of feature analysis in K562 and Hela cells are consistent with those in GM12878.

(**a**) Ranking of the predictive importance of the top 20 features in the model trained in HeLa and K562.

(**b**) Distributions of the average binding intensity of CTCF and RAD21 on anchors in negative and positive loops in the three cell lines. *P*-values were calculated using Mann-Whitney U test.

(**c**) Distributions of the intensity of the indicated histone marks within negative and positive loops. *P*-values were calculated using Mann-Whitney U test.

(**d**) Distributions of relative fluctuations of CTCF and RAD21's binding intensities on paired anchors of negative and positive loops in HeLa and K562 cells. Relative fluctuation was defined as the ratio of standard deviation to average value. *P*-values were calculated using Mann-Whitney U test.

(e) Ranking of the predictive importance of the top 20 features in wiring prediction. The model was trained in the rewiring data (see methods for details) of GM12878, HeLa and K562 cells, respectively.

(**f**) Heatmaps of feature correlation in HeLa and K562 cells.

(**g**) Comparison of model performance in GM12878 using PR curves. CTCF motif-only features include motif orientation pattern, motif strength and conservation score on anchors. ChIP-seq features include CTCF binding strength at the two anchors. Features without CTCF and RAD21 denotes all features except those derived from CTCF and RAD21 ChIP-seq.

Supplementary Figure 4

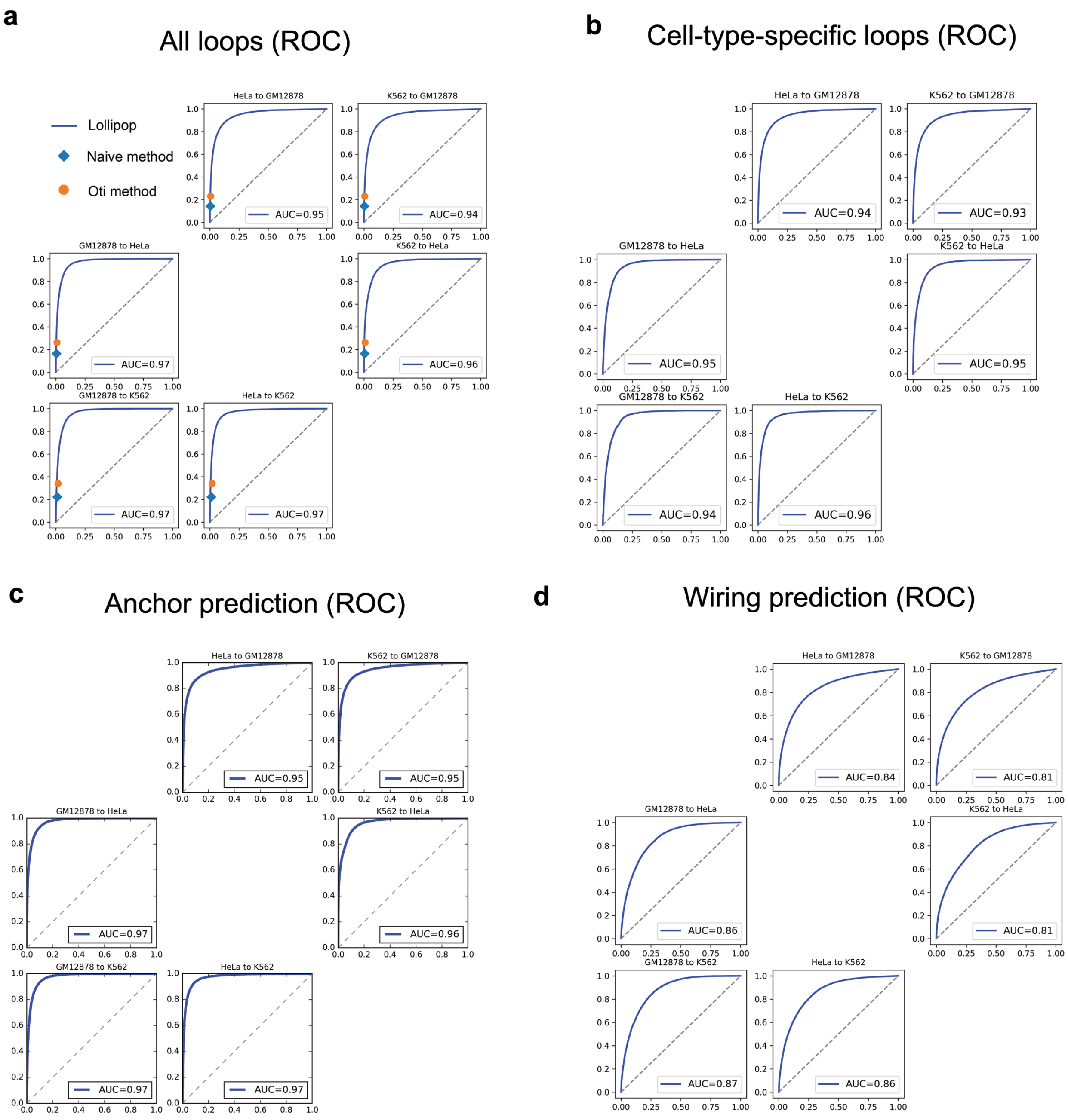

Supplementary Figure 4. Performance evaluation of Lollipop across cell-types.

(**a**) Across-cell-type performance evaluation using ROC curves.

(**b**) Across-cell-type performance evaluation on cell-type-specific loops using ROC curves.

(**c**) Performance evaluation of anchor prediction using ROC curves.

(**d**) Performance evaluation of wiring prediction using ROC curves.

Supplementary Figure 5

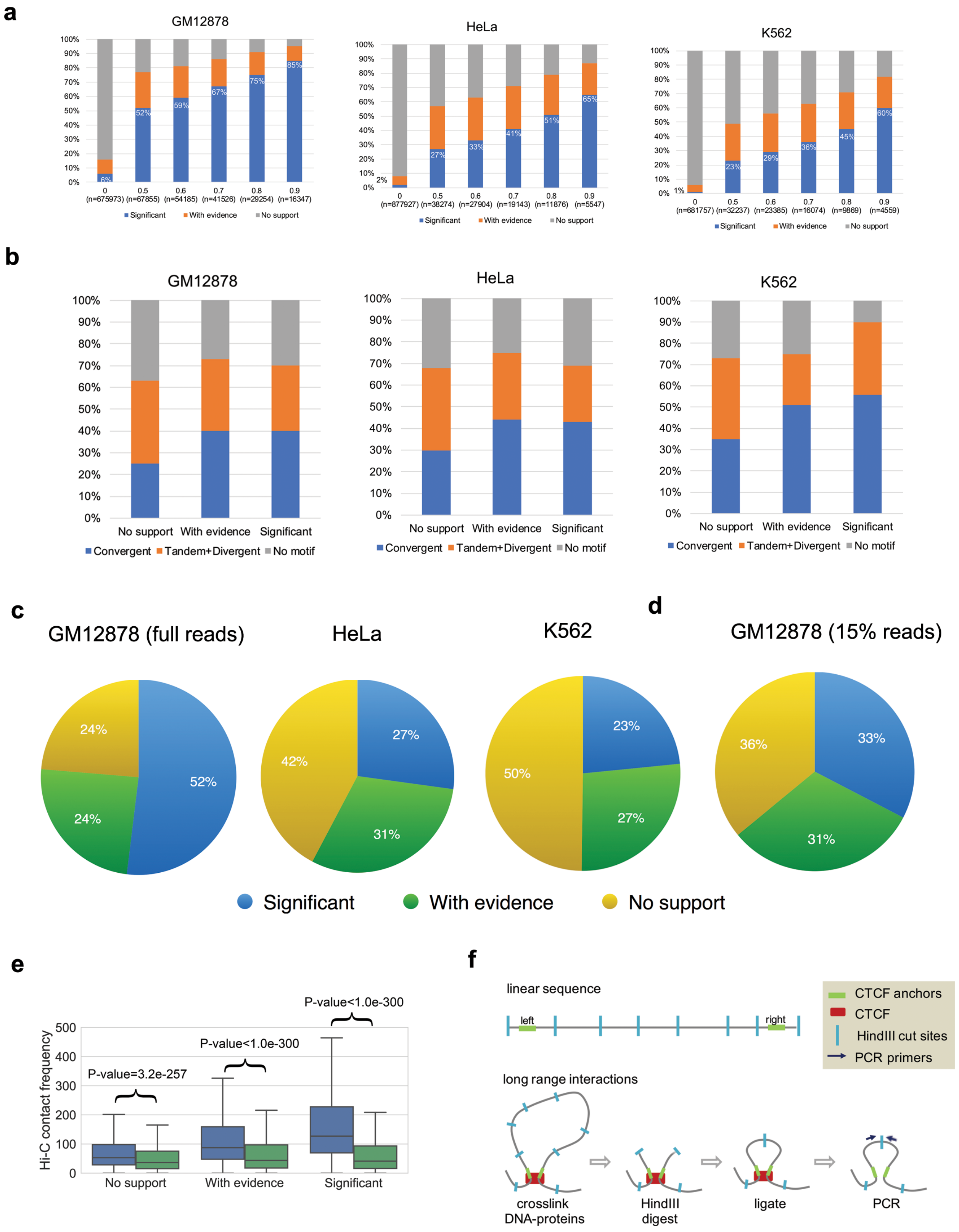

g

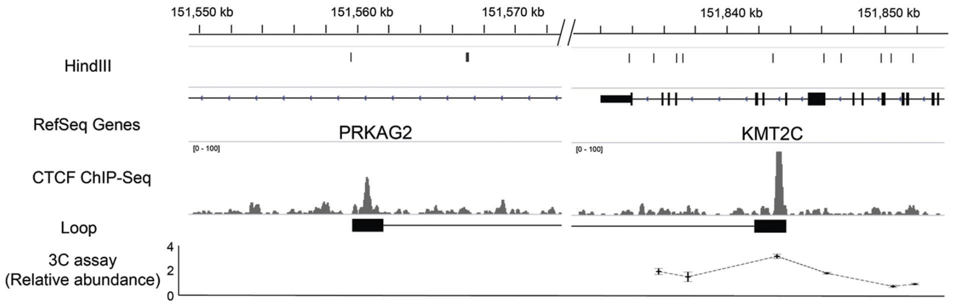

151,550 kb
151,560 kb
151,570 kb
151,840 kb
151,850 kb
HindIII
RefSeq Genes
PRKAG2
KMT2C
CTCF ChIP-Seq
[0 - 100]
[0 - 100]
Loop
3C assay
(Relative abundance)
4
2
0

h

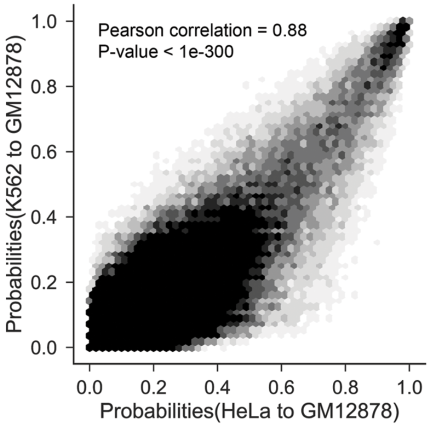

Pearson correlation = 0.88
P-value < 1e-300
Probabilities(K562 to GM12878)
Probabilities(HeLa to GM12878)
0.0
0.2
0.4
0.6
0.8
1.0

i

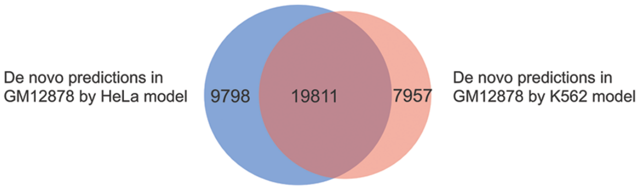

De novo predictions in
GM12878 by HeLa model
9798
19811
7957
De novo predictions in
GM12878 by K562 model

Supplementary Figure 5. Validation of predicted CTCF-mediated interactions.

(**a**) Comparison of *de novo* predicted loops with ChIA-PET data at varying prediction stringencies. The prediction stringency (probability cut-off value) and the number of predicted loops are denoted at the bottom of each column. The first column shows the expected overlap in background.

(**b**) The distribution of motif orientation patterns of loops in the “No support”, “With evidence” and “Significant” categories. Loops in the ‘With evidence’ and ‘Significant’ categories are enriched with convergent motif orientation compared with loops in the ’No support’ category (*P*-value < 1e-100 for all pair-wise comparison, Chi-squared test).

(**c**) The distribution of *de novo* predicted loops as compared to the ChIA-PET data. 0.5 was chosen as the probability cut-off value.

(**d**) The distribution of predicted loops using a down-sampled (to 15%) GM12878 library for model building, followed by genome-wide prediction and comparison with ChIA-PET data. The number of loops observed in the downscaled GM12878 library is similar to those of K562 and HeLa (see **Supplementary Table 2**).

(**e**) Hi-C validation of “No support”, “With evidence” and “Significant” loops in GM12878. Blue, predicted loops. Green, random genomic regions with matching size and length distribution. Loops with stronger support from ChIA-PET have higher contact frequency in Hi-C (*P*-value <1e-300 for both “With evidence” vs. “No support” and “Significant” vs. “With evidence”). *P*-values were calculated using Mann Whitney U test.

(**f**) Illustration demonstrating the major steps of 3C experiments.

(**g**) 3C-qPCR analysis shows the relative abundance of *PRKAG2* anchor to *KMT2C* anchor and adjacent HindIII fragments (**Fig. 6b** top panel). Tracks from top to bottom: HindIII cut sites, designed primer for testing interaction, CTCF ChIP-Seq, motif occurrences, and relative quantification of the 3C interaction.

(**h**) Scatter plot with hexagonal binning showing the looping probabilities of all potential loops in GM12878 predicted from from HeLa (x-axis) and K562 (y-axis) models.

(**i**) The overlap of loops in GM12878 predicted from HeLa and K562 models. The overlap is significant (*P*-value < 1e-300, hypergeometric test).

Supplementary Figure 6

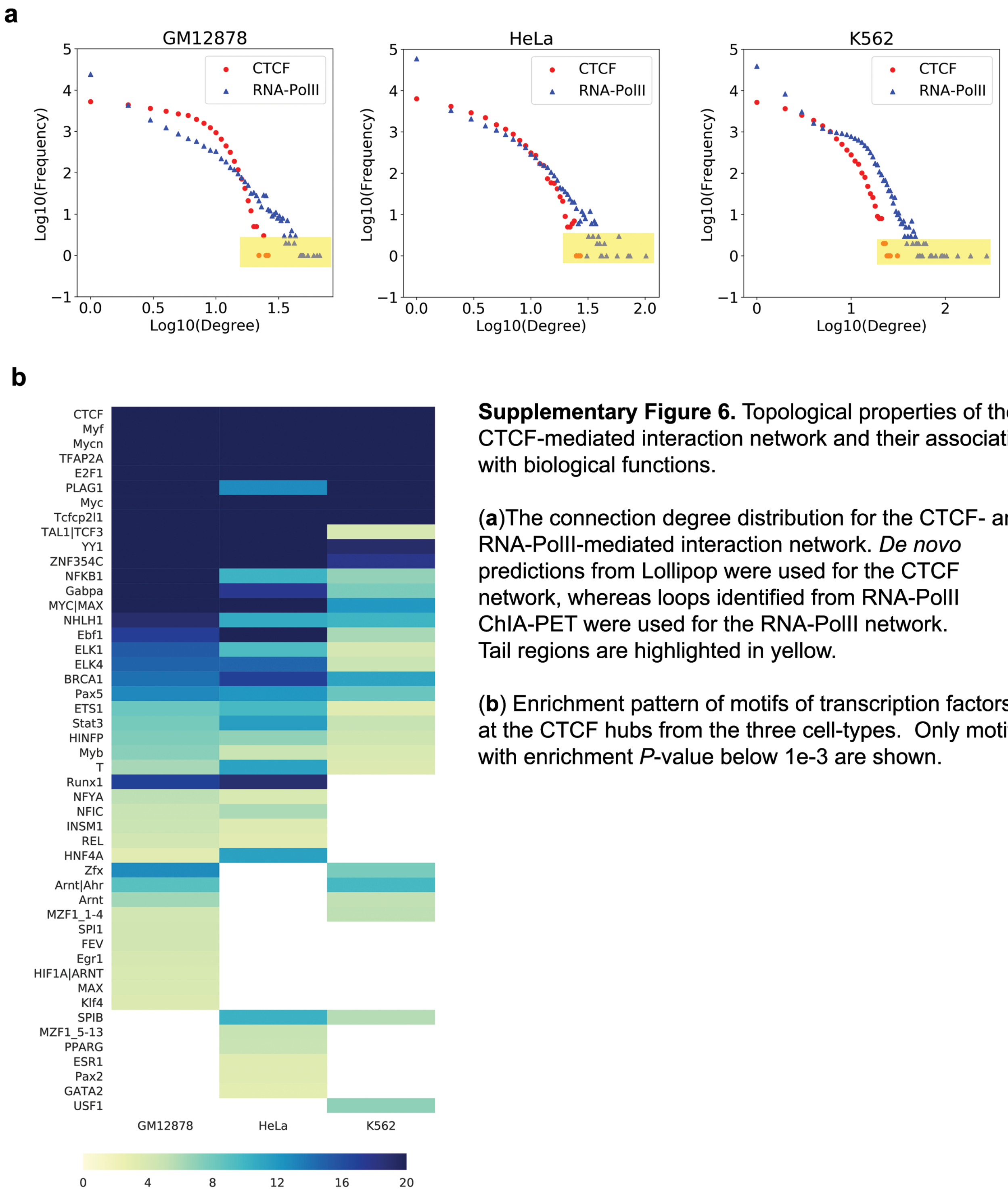

Supplementary Figure 6. Topological properties of the CTCF-mediated interaction network and their association with biological functions.

(**a**)The connection degree distribution for the CTCF- and RNA-PolII-mediated interaction network. *De novo* predictions from Lollipop were used for the CTCF network, whereas loops identified from RNA-PolII ChIA-PET were used for the RNA-PolII network. Tail regions are highlighted in yellow.

(**b**) Enrichment pattern of motifs of transcription factors at the CTCF hubs from the three cell-types. Only motifs with enrichment *P*-value below 1e-3 are shown.

Supplementary Table 1: Data sets used

Data	GM12878	HeLa	K562
ChIA-PET	GSE72816[4]	GSE72816[4]	ENCLB559JAA[1,2]
Hi-C	GSE63525[3]		GSE63525[3]
DNase-Seq	ENCFF000SKV[1,2]	ENCFF000SPJ[1,2]	ENCFF000SVI[1,2]
RNA-Seq	ENCFF000FBU[1,2]<br>ENCFF000FBV[1,2]	ENCFF158RCK[1,2]<br>ENCFF169ZTB[1,2]	GSM765393[1,2]
ChIP-Seq (CTCF)	ENCFF000ARG[1,2]	ENCFF000BAJ[1,2]	ENCFF000YLT[1,2]
ChIP-Seq (RAD21)	ENCFF000OBV[1,2]	ENCFF000XKH[1,2]	ENCFF084HTD[1,2]
ChIP-Seq (H2AZ)	ENCFF001SUD[1,2]	ENCFF000BAX[1,2]	ENCFF000BWO[1,2]
ChIP-Seq (H3K4me1)	ENCFF000ARY[1,2]	ENCFF000BBA[1,2]	ENCFF000BXK[1,2]
ChIP-Seq (H3K4me2)	ENCFF000ATG[1,2]	ENCFF000BCH[1,2]	ENCFF000BXT[1,2]
ChIP-Seq (H3K4me3)	ENCFF000ATS[1,2]	ENCFF000BCO[1,2]	ENCFF000BXW[1,2]
ChIP-Seq (H3K9ac)	ENCFF000ATY[1,2]	ENCFF000BCW[1,2]	ENCFF000BYK[1,2]
ChIP-Seq (H3K9me3)	ENCFF000AUH[1,2]	ENCFF000BBG[1,2]	ENCFF000BYT[1,2]
ChIP-Seq (H3K27ac)	ENCFF000ASI[1,2]	ENCFF000BBN[1,2]	ENCFF000BWZ[1,2]
ChIP-Seq (H3K27me3)	ENCFF000ASK[1,2]	ENCFF000BBS[1,2]	ENCFF000BXA[1,2]
ChIP-Seq (H3K36me3)	ENCFF000ASX[1,2]	ENCFF000BCC[1,2]	ENCFF000BXE[1,2]
ChIP-Seq (H3K79me2)	ENCFF000ATT[1,2]	ENCFF000BCQ[1,2]	ENCFF000BYC[1,2]
ChIP-Seq (H4K20me1)	ENCFF000AUT[1,2]	ENCFF000BDC[1,2]	ENCFF001QWY[1,2]
ChIP-Seq Input	ENCFF000AQZ[1,2]	ENCFF000BAI[1,2]	ENCFF000BVZ[1,2]
ChIP-Seq Input	ENCFF651WEV[1,2]	ENCFF469INX[1,2]	ENCFF000QEK[1,2]

Supplementary Table 2: Analysis results of ChIA-PET data sets

Cell-type	Raw reads (in million)	Unique PETs (in million)	IAB >= 2 loops	FDR <= 0.05 loops	IAB >=2 and FDR <= 0.05 loops
GM12878 (full reads)	680	39.8	93914	73511	51966
GM12878 (15% reads)	102	13.1	37125	22248	15569
HeLa	531	21.1	42430	25047	16783
K562	195	6.6	23884	23377	13076

Supplementary Table 3: Top-ranked features from the Recursive Feature Elimination analysis

*Numbers inside the parentheses indicate the times of top-ranked feature set appears.
*'avg' and 'std' represent the mean and standard deviation of the signal intensity on both anchors. '_left' and '_right' represent the flanking features while '_in-between' means the signal intensity in the loop region.

	GM12878
**Top 1 feature**	avg_RAD21 (5)
**Top 2 features**	avg_CTCF, avg_RAD21 (3)<br>motif_pattern, avg_RAD21 (2)
**Top 4 features**	length, motif_pattern, avg_CTCF, avg_RAD21 (5)
**Top 8 features**	length,motif_pattern,avg_motif_strength,HS_in-between,avg_CTCF,std_CTCF,CTCF_in-between,avg_RAD21 (4)<br>length,motif_pattern,avg_motif_strength,avg_CTCF,std_CTCF,CTCF_in-between,avg_RAD21,std_RAD21 (1)
**Top 16 features**	length,motif_pattern,avg_motif_strength,HS_in-between,avg_H3K4me1,avg_H3K27ac,H4K20me1_in-between,avg_CTCF,std_CTCF,CTCF_in-between,CTCF_left,CTCF_right,avg_RAD21,std_RAD21,RAD21_in-between,RAD21_left (2)<br>length,motif_pattern,avg_motif_strength,HS_in-between,avg_H3K4me1,avg_H3K27ac,avg_CTCF,std_CTCF,CTCF_in-between,CTCF_left,CTCF_right,avg_RAD21,std_RAD21,RAD21_in-between,RAD21_left,expression (3)
	**HeLa**
**Top 1 feature**	avg_RAD21 (5)
**Top 2 features**	length, avg_RAD21 (5)
**Top 4 features**	length, motif_pattern, avg_CTCF, avg_RAD21 (5)
**Top 8 features**	length,motif_pattern,avg_motif_strength,avg_H3K4me1,avg_CTCF,CTCF_in-between,avg_RAD21,std_RAD21 (5)
**Top 16 features**	length,motif_pattern,avg_motif_strength,avg_HS,avg_H3K4me1,H3K4me3_in-between,avg_CTCF,std_CTCF,CTCF_in-between,CTCF_right,avg_RAD21,std_RAD21,RAD21_in-between,RAD21_left,RAD21_right,expression (1)<br>length,motif_pattern,avg_motif_strength,std_motif_strength,avg_H3K4me1,H3K4me3_in-between,avg_CTCF,std_CTCF,CTCF_in-between,CTCF_left,CTCF_right,avg_RAD21,std_RAD21,RAD21_in-between,RAD21_left,RAD21_right (4)
	**K562**
**Top 1 feature**	avg_CTCF (1)<br>avg_RAD21 (4)
**Top 2 features**	length, avg_CTCF (1)<br>avg_CTCF, avg_RAD21 (3)<br>length, avg_RAD21 (1)
**Top 4 features**	length,avg_CTCF,CTCF_left,avg_RAD21(5)
**Top 8 features**	length,motif_pattern,avg_CTCF,std_CTCF,CTCF_in-between,CTCF_left,avg_RAD21,RAD21_left (5)
**Top 16 features**	length,motif_pattern,avg_motif_strength,std_HS,HS_in-between,HS_left,avg_CTCF,std_CTCF,CTCF_in-between,CTCF_left,CTCF_right,avg_RAD21,std_RAD21,RAD21_in-between,RAD21_left,expression (4)<br>length,motif_pattern,avg_motif_strength,HS_in-between,HS_left,H3K4me3_in-between,avg_CTCF,std_CTCF,CTCF_in-between,CTCF_left,CTCF_right,avg_RAD21,std_RAD21,RAD21_in-between,RAD21_left,expression (1)

Supplementary Table 4: Designed primers for 3C validation

Primer Name	Sequence (5’ to 3’)
KMT2C_U2	FGGAGAGGATGATGGTGCTGTGTAT
KMT2C_U1	CTTGATCGTTTCTCACTCCTTTCA
KMT2C_L	CTTGACTGTCACCTTCAGCTCATC
KMT2C_D1	GACATACCAGAGCAATAACCTGGA
KMT2C_D3	AGCAGCAAATGAATCAGCTCAG
KMT2C_D4	AGTGGTGTCAATGCTGGTTTTC
KMT2C_R	ATCACTGTCTAGCTGCCCGTTC
PDGFRB_L	TATGCAGTGGTTTGTACCCTTG
PDGFRB_R	GTGGCACCATAATCATCCCTAT

Supplementary References

[1]Consortium, E. P. (2012). "An integrated encyclopedia of DNA elements in the human genome." Nature **489**(7414): 57-74.

[2]Sloan, C. A., E. T. Chan, J. M. Davidson, V. S. Malladi, J. S. Strattan, B. C. Hitz, I. Gabdank, A. K. Narayanan, M. Ho, B. T. Lee, L. D. Rowe, T. R. Dreszer, G. Roe, N. R. Podduturi, F. Tanaka, E. L. Hong and J. M. Cherry (2016). "ENCODE data at the ENCODE portal." Nucleic Acids Res **44**(D1): D726-732.

[3]Rao, S. S., M. H. Huntley, N. C. Durand, E. K. Stamenova, I. D. Bochkov, J. T. Robinson, A. L. Sanborn, I. Machol, A. D. Omer, E. S. Lander and E. L. Aiden (2014). "A 3D map of the human genome at kilobase resolution reveals principles of chromatin looping." Cell **159**(7): 1665-1680.

[4]Tang, Z., O. J. Luo, X. Li, M. Zheng, J. J. Zhu, P. Szalaj, P. Trzaskoma, A. Magalska, J. Wlodarczyk, B. Ruszczycki, P. Michalski, E. Piecuch, P. Wang, D. Wang, S. Z. Tian, M. Penrad-Mobayed, L. M. Sachs, X. Ruan, C. L. Wei, E. T. Liu, G. M. Wilczynski, D. Plewczynski, G. Li and Y. Ruan (2015). "CTCF-Mediated Human 3D Genome Architecture Reveals Chromatin Topology for Transcription." Cell **163**(7): 1611-1627.
